# Bioacoustic Field Research: A Primer to Acoustic Analyses and Playback Experiments With Primates

**DOI:** 10.1002/ajp.22153

**Published:** 2013-04-16

**Authors:** JULIA FISCHER, RAHEL NOSER, KURT HAMMERSCHMIDT

**Affiliations:** Cognitive Ethology Laboratory, German Primate CenterGöttingen, Germany

**Keywords:** acoustic analysis, alarm calls, cognition, communication, playback experiments

## Abstract

Acoustic analyses of primate vocalizations as well as playback experiments are staple methods in primatology. Acoustic analyses have been used to investigate the influence of factors such as individuality, context, sex, age, and size on variation in calls. More recent studies have expanded our knowledge on the effects of phylogenetic relatedness and the structure of primate vocal repertoires in general. Complementary playback experiments allow direct testing of hypotheses regarding the attribution of meaning to calls, the cognitive mechanisms underpinning responses, and/or the adaptive value of primate behavior. After briefly touching on the historical background of this field of research, we first provide an introduction to recording primate vocalizations and discuss different approaches to describe primate calls in terms of their temporal and spectral properties. Second, we present a tutorial regarding the preparation, execution, and interpretation of field playback experiments, including a review of studies that have used such approaches to investigate the responses to acoustic variation in calls including the integration of contextual and acoustic information, recognition of kin and social relationships, and social knowledge. Based on the review of the literature and our own experience, we make a number of recommendations regarding the most common problems and pitfalls. The power of acoustic analyses typically hinges on the quality of the recordings and the number of individuals represented in the sample. Playback experiments require profound knowledge of the natural behavior of the animals for solid interpretation; experiments should be conducted sparingly, to avoid habituation of the subjects to the occurrence of the calls; experimenter-blind designs chosen whenever possible; and researchers should brace themselves for long periods of waiting times until the appropriate moments to do the experiment arise. If all these aspects are considered, acoustic analyses and field playback experiments provide unique insights into primate communication and cognition. Am. J. Primatol. 75:643–663, 2013. © 2013 Wiley Periodicals, Inc.

## BACKGROUND

Richard Garner was probably unaware that he had applied some of the most powerful and influential methodologies to investigate primate communication and cognition when, in 1890, he used Edison's recently patented “speaking machine,” a version of the phonograph, to record the calls of capuchin and rhesus monkeys and to play them back to conspecifics in the Zoological gardens of Washington and New York [reviewed in Radick, 2008]. At that time, modern ethology was yet to be established by figures like Niko Tinbergen and Konrad Lorenz, and this may be the reason why Garner's pioneering work, retrospectively lacking many important qualities of modern ethological research, simply was forgotten.

In the twentieth century, the study of vocal communication in both birds and primates was spearheaded by Peter Marler, who had studied in Britain and then moved first to Rockefeller University at the United States' East Coast, and later to the University of California at Davis. In 1955, he had published a note on the “Characteristics of some Animal Calls” in *Nature* [Marler, 1955], and later, in 1968, a treatise on the “Vocalizations of Wild Chimpanzees” [Marler, 1969]. It was Struhsaker's [1967] study on vocal behavior of vervet monkeys*, Chlorocebus pygerythrus*, then classified as *Cercopithecus aethiops*, however, that marked the turning point in the analysis in free ranging primates: Struhsaker had tape-recorded and spectrographically analyzed a total of 64 hr of vervet monkey calls, and had carefully described their acoustical, contextual, and functional properties. What he found was intriguing: at least 36 spectrographically and audibly different sounds, by which according to him at least 21 messages were communicated and which evoked at least 18 different, seemingly adaptive responses [Struhsaker, 1967]. Notably, this careful piece of work suggested that the vervet alarm calls differed according to the nature of danger, namely snakes, mammalian, or avian predators. These findings opened the way to the idea that animal alarm calls may designate external objects or events, rather than pure internal motivational states.

These observations inspired Peter Marler to investigate the issue further. He sent two postdoctoral fellows—Dorothy Cheney and Robert Seyfarth, who had just completed their PhDs with Robert Hinde at Cambridge—to Amboseli to study the function and meaning of vervet monkey alarm calls using playback experiments. This time, the potential of this methodology to investigate primate minds was immediately acknowledged. The publications stemming from 14 months of fieldwork [Seyfarth et al., 1980a, 1980b] were influential in several ways: they established acoustical playback experiments as a powerful methodological tool in primatology; they provided the first direct evidence that the primate calls are individually distinct [Cheney & Seyfarth, 1980]; and most importantly, they revealed that the calls alone are sufficient to elicit the appropriate responses in listeners, even in the absence of the predator.

### Purpose of This Paper

Analyses of the structure of primate vocalizations as well as playback experiments are complementary and staple methods for understanding primate communication. Although bioacoustic research may appear simple at first glance, there are indeed many pitfalls that may turn out to be fatal for a study. The purpose of the present paper is to review and discuss some key issues regarding firstly the recording and analysis of primate vocalizations, and secondly playback experiments. We outline important issues that need to be considered during the planning stage of a study, discuss technical aspects important for obtaining high-quality recordings, provide a brief tutorial regarding the different features that can be measured, and review some of the most frequently used statistical tools used in acoustic analyses. We hope to make the case that—if done carefully and properly—acoustic analyses can provide important insights into the factors that contribute to variation in acoustic signals and signal design more generally. In the second part of this paper, we take on the design and execution of playback studies. We use various worked examples to show how the research question drives the choice of the experimental design. This review section documents that playback experiments may not only be used to test hypotheses from acoustic analyses but also as a tool to elucidate what animals know about each others' relationships, and how they make decisions. In this section, we also consider statistical issues. This review is aimed at novices in the field, as well as more experienced researchers, as a reference. We hope that this contribution will help others to avoid some of the mistakes that we (and others) made in the past, and that it will promote future substantive and sound studies.

## ACOUSTIC ANALYSES

### Planning the Study

As with every rigorous piece of research, planning is essential. Too often, however, we have witnessed that too little attention is given to this stage, so that researchers are coming back sometimes from months of hardship under demanding field conditions, with data that are not suited to address their research question. We therefore strongly recommend investing sufficient time into the planning of the study. Firstly, one needs to think carefully which data are needed to address the research question and test one's hypotheses. Given the circumstances and available resources (time and budget), will it be reasonably certain that one will be able to collect these data? For instance, one needs to clarify whether there are sufficient animals in the area, and perhaps whether they are sufficiently habituated to allow for collecting high-quality recordings. Will it be possible to conduct the experiments at a rate that does not lead to habituation of the subjects to the experimental situation, and so on. It is also important at this stage to think about the statistical analyses that will be applied to the data.

To some degree, the data-collection scheme depends on whether the study is more exploratory/descriptive, or aims at testing specific hypotheses. In the latter case, more stringent criteria apply. But even if one simply aims to address the question whether, say, male and female calls differ, it is crucial to consider the sample size necessary to make meaningful inferences. Likewise, if one aims to test the hypothesis whether male calls are related to fighting ability, a sufficient number of subjects that exhibit some variation in quality are required. As noted above, this all may sound trivial, but we have seen too many examples where such essential aspects were only considered after completion of the data collection.

#### Statistical power

As a rule of thumb, we recommend to maximize the number of subjects whose calls are recorded, as this is decisive for the statistical power of the analysis (see “Statistical analysis”). The number of calls per subject mainly depends on the research question. In principle, one call can be sufficient, but since primate vocalizations often show substantial intra-individual variation, it is a good idea to use several calls per individual. There are no hard and fast rules on how many calls should be recorded. The sample size needed depends on the intra-individual variation present in the call type under consideration, which can vary substantially. The larger the variation, the more calls are needed per individual. In our experience, using 20 calls per individual sufficiently addresses the possible impact of intra-individual variation in calling, but a lower number may be sufficient. If calls are given in bouts, one should extract one call per bout for later analysis. Ideally, one should assess the intra-individual variation and the stability of statistical analyses in a pilot phase by running analyses with different subsets of calls. If the results are stable, the number of calls per subject is likely to be sufficient.

#### Choice of variables

Generally speaking, if the study is hypothesis driven, one should select the acoustic variables of interest prior to their extraction, based on the theoretical background. For instance, in a study in which we tested the hypothesis that male baboon loud calls are related to their fighting ability, we focused on a small number of variables for which we could make specific predictions [Fischer et al., 2004]. In a study on the relationship between acoustic characteristics and timing of ovulation in female Barbary macaques, we had predicted that changes in tissue water content should lead to changes in the regularity of vocal fold oscillation, and thus jitter and shimmer [Pfefferle et al., 2011]. This focus on specific variables reduces problems associated with multiple testing of the same general hypothesis, which requires an adjustment of the p-levels. With exploratory analyses, various parameters can be assessed. Sometimes, it is not possible to formulate specific predictions, for instance, when one is interested in individual differences in calling, where it may be difficult to predict the type of variation that can be found. One should, however, always be aware of data dredging that is using the same dataset to generate the hypotheses and then to test them. This is not permissible.

### Equipment

#### Recorders

The current system of our choice are digital solid-state recorders, and we have never experienced any problems with them, even in the rain forest of Siberut Island [Schneider et al., 2008] or the dust and heat of the Kalahari desert [Meise et al., 2011], when care is taken to store them appropriately. There are several recorder models available and depending on one's requirements and budget, one will typically try to balance technical finesse, robustness, weight, and price. Large jacks or microphone jacks are generally more robust than small jacks. Some solid-state recorders have a feature that comes in very handy for field recordings, namely a buffer (“pre-record”), which provides the possibility to continuously record 2 sec (for instance), which are saved once you press “record,” and otherwise discarded. As animals frequently burst out in vocalizations unexpectedly, this allows you to save the beginning of a bout of calls as well, without the need of continuous recordings followed by screening through hours of more or less silent audio files.

#### Microphones

The use of a proper microphone is a further prerequisite for good field recordings. For recording calls from individual subjects, it is indispensable to use a professional directional microphone. A frequently used combination in bioacoustics is the Sennheiser K6 power module with a ME66 recording head. For recording low-pitched sounds over larger distances, Sennheiser offers a longer microphone head (ME67). Other brands may be of similar quality, but the use of cheap consumer electronics microphones is strongly discouraged. Microphone holders are useful to avoid disturbing noises by hand movements on the microphone, but with sufficient training and discipline, one may work without them.

#### Windshields

The microphone head must be protected by a windshield or windscreen. Its size and quality depend on the recording conditions. Small foam windshields can be used for recordings in buildings or under fine outside conditions. More windy conditions require stronger protection, such as the Rycote softie windshield (Rycote, Gloucestershire, UK), which combines foam with an integral fur cover. If such a protection is not sufficient, one may opt for a modular windshield with microphone suspension, where the microphone is completely surrounded by a basket and protected with a second layer (e.g., Rycote Windjammer). Some researchers prefer to work with two microphones, a directional one that is used to record the animal's vocalizations, and a second one that is clipped to the collar, to record one's comments.

### Sampling Accuracy and Sampling Rate

The quality of the (digital) recording and thus the basis for the analysis depends crucially on the sampling accuracy and the sampling rate. Generally, it is recommended to go for 16 bits of storage depth, which provides a higher accuracy than using 8 bits. Given the average quality of field recordings, 24 bit sampling depth does not add much to the quality, however. The use of compression formats such as MP3 is discouraged, as the signal will be compressed and modified; go for CD-quality (e.g., PCM-WAV file format) instead. The sampling rate is also decisive for the precision with which the continuous analogue change in the sound pressure wave is turned into a digital representation. The more sampling points per unit time, the better the representation of the analogue signal, and the higher the temporal resolution. A commercial CD uses a sampling frequency of 44.1 kHz; but many recorders also offer 48 kHz or even 96 kHz sampling frequency. Researchers interested in ultrasonic vocalizations need to use even higher sampling frequencies, as the sampling frequency limits the frequency range that can be analyzed. For digitization, a wave must be sampled at least twice before its frequency can be determined with sufficient accuracy. Therefore, one needs to select at least twice the frequency of the highest frequency in the sound (the so-called Nyquist frequency). If the sound spectrum contains energy above the Nyquist frequency, this may lead to artifacts known as “aliasing.” Therefore, a filter that removes all energy above the Nyquist frequency before digitization has to be applied when using analogue tapes. Recently, digital recorders have in-built filters. In sum, one needs to select the appropriate sampling frequency, but one should not aim to go higher than needed, because there is a trade-off between sampling frequency and frequency resolution in the spectral analysis (see below).

### Recording in the Field

#### Quality control

The novice should take sufficient time to become familiar with the equipment, and to check the recordings, in order to ensure that the verbal commentary is on the one hand sufficiently precise to later classify the calls according to ID, context, etc., but on the other hand timed in such a way that the calls themselves are not disturbed. Ideally, one wants to place the commentary in between calls or call bouts, but this is sometimes tricky. One should plan sufficient time to figure out how to balance these two different interests in an efficient manner. When calls are high pitched, the rather low frequency comments by the observer may not be such a nuisance, but observer commentary renders low frequency calls unsuitable for further analysis. Therefore, it is essential not only to listen to your own recordings shortly after recording but also to monitor the quality by looking at the real-time spectrogram (if possible) while listening (e.g., Adobe Audition [Adobe Systems, Inc., San Jose, CA] or Avisoft [R. Specht, Berlin, Germany]). Sometimes, the disastrous influence of your own hand movements and the sounds of your steps only become apparent after the fact. Also, one needs to develop a feel for the impact of background noise such as leaves rustling in the wind, birds singing, or insects chirping. The ultimate goal is to collect recordings with a high signal-to-noise ratio (large difference between signal amplitude and the amplitude of other sound sources), and no appreciable disturbances by other animals' calling. This is particularly important if you aim for automated extraction of variables by specific software. If you confine yourself to the inspection of spectrograms or temporal features, then lower quality recordings may also be used.

#### Recording distance

In our experience, one important aspect regarding the recording quality is the distance between the animal that is calling and the microphone. The attenuation of the signal during transmission is frequency dependent, with higher frequencies attenuating more strongly than lower frequencies. In addition, the habitat characteristics, height of the sound source, and further factors such as humidity and wind speed all affect attenuation and reverberation [Bradbury & Vehrencamp, 2011; Wiley & Richards, 1978]. The detrimental influence of increasing distance was tested by Maciej et al. [2011], who re-recorded a set of different baboon calls at different distances, at different heights, and in different habitats. They found that higher energy components are disproportionately affected by increasing recording distance. The impact of increasing distance depended strongly on the call type. High amplitude tonal calls, in which the energy is confined to a small frequency band, were less susceptible to degradation than atonal and noisy calls, for instance. Therefore, it is advisable to develop some feel for the degree of degradation that the calls of interest are experiencing. For low amplitude calls such as baboon grunts, one may want to limit oneself to the analysis of calls recorded at no more than 3–5 m, while high amplitude calls may still yield replicable measurements at distances of up to 15 m, depending on the structure.

#### Annotating recordings

Once the desired vocalizations are recorded, they are transferred to the computer. In many cases, a file will contain several call bouts, which in turn may contain many calls. In our lab, we are applying a naming procedure where each call element can be traced back to the call bout it came from, as well as the original file, by using a hierarchical naming system. Others prefer to code caller ID or call type in the file name; whatever you choose, it is important to be able to trace back every recording to its original context. This is essential to reconfirm other contextual information. The “master file” should be well annotated, including information on who made the recording, the equipment used, the recording distance, and so on (Table 1). In times where large data repositories are becoming the norm, such meta-data are absolutely crucial for broader analyses.

**Table I:** Information that Should Be Included When Annotating Recordings

General category	Specific information
Equipment	Recorder, microphone, windscreen (brands and types)
Recorder settings	File format, sampling frequency, sampling accuracy, recording level
Recording conditions	Weather conditions, habitat structure, subject distance
Subject information	Individual identity, age, sex, behavioral context (including other subjects' behavior)
General information	Who made the recordings, location (GPS points), time, study group

### Measuring Acoustic Features

#### Temporal features

Measurements of acoustic features can be made regarding both temporal and spectral characteristics of the call. If one is mainly interested in temporal aspects, measurements can be made with the aid of a cursor from the waveform, using a spectrographic depiction (see below) to confirm the quality of the calls. Call rate, number of elements in a calls, etc., can relatively easily be assessed in this way. It is, however, advisable to use software to automatically extract the features, because in this way, one is forced to set clear criteria for the demarcation of different elements or the on- and off-set of calls. This ensures replicable measurements. Tried and tested software includes freely available packages such as Praat (praat.org), commercial software such as Avisoft (Raimund Specht, Berlin), RAVEN (Cornell Lab of Ornithology), or SIGNAL (Kim Beeman, Engineering Design). If automated extraction is not feasible, then it is essential to check both intra- and inter-observer reliability.

#### Power spectra and Fourier transforms

**Fig. 1:**
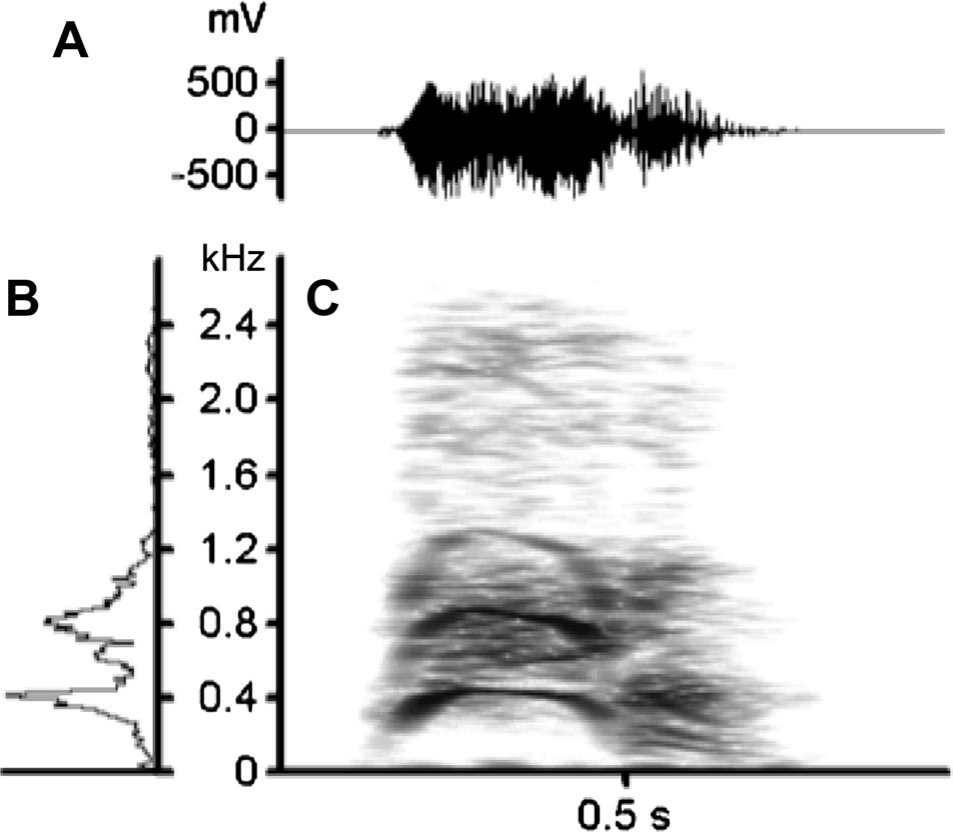
Male baboon clear bark (“wahoo”): A: waveform (envelope) depicting the amplitude variation over time. B: Power spectrum, showing the distribution of the amplitude in the frequency spectrum, here mean (normalized) across the entire call. C: Spectrogram, depicting the distribution of the amplitude (different shades of gray) across the frequency spectrum and over time. Note that (B) and (C) share the same y-axis. FFT length = 512, Hamming window, overlap 96.9%, sampling frequency = 5.5 kHz, time resolution = 2.9 msec.

To characterize the spectral characteristics of a signal, power spectra, and spectrograms are used. Power spectra depict the energy distribution over the frequency spectrum; spectrograms illustrate the amplitude distribution over frequency and time (Fig. 1). The spectral analysis rests on Fourier's theorem according to which each complex waveform can be decomposed into a set of pure sine waves. Spectral analyses are constrained by the fact that time resolution and frequency resolution are inversely related (time resolution × frequency resolution = 1). Thus, if the frequency resolution needs to be high, the analyzed segment must be sufficiently long. Conversely, if the temporal resolution is high, spectral features are blurred. A signal with a sampling rate of 44.1 kHz subjected to a 1,024-pt FFT (Fast Fourier Transform) will yield a frequency resolution of ∼43 Hz. With such a frequency resolution it is impossible to estimate the fundamental frequency of low-pitched vocalizations like baboon grunts. The fundamental frequency of a male baboon grunts can be less than 80 Hz. Thus, it is advisable to downsample the signal, in case of male grunts to 5.5 kHz (or lower), enabling a frequency resolution of ∼5.5 Hz (or more), given an FFT length of 1,024-pt. The temporal resolution can be artificially increased by “sliding” the transformation window across the entire signal in very small steps that is with some overlap. In this case, a weighting function is used that emphasizes the center of the window, and de-emphasizes the edges, such as a Hanning or a Hamming window. Further details can be found in the literature [e.g., Kent & Read, 1992].

### Extraction of Acoustic Features

There are two general approaches to measure different aspects of the sound. One is based on extracting features from the spectrograms, being agnostic about the call production mechanisms. Another approach that is frequently used is “linear predictive coding” or LPC. LPC analyses are driven by theoretical assumptions regarding vocal production, namely the source-filter model, according to which sounds are produced by oscillations of the vocal folds, which then pass through the vocal tract that acts as a filter such that specific frequency ranges are diminished while others pass through. These are called “formants” [Fitch, 2000]. Hence, LPC analyses are also known as “formant analyses.”

#### Spectrogram-based features

**Fig. 2:**
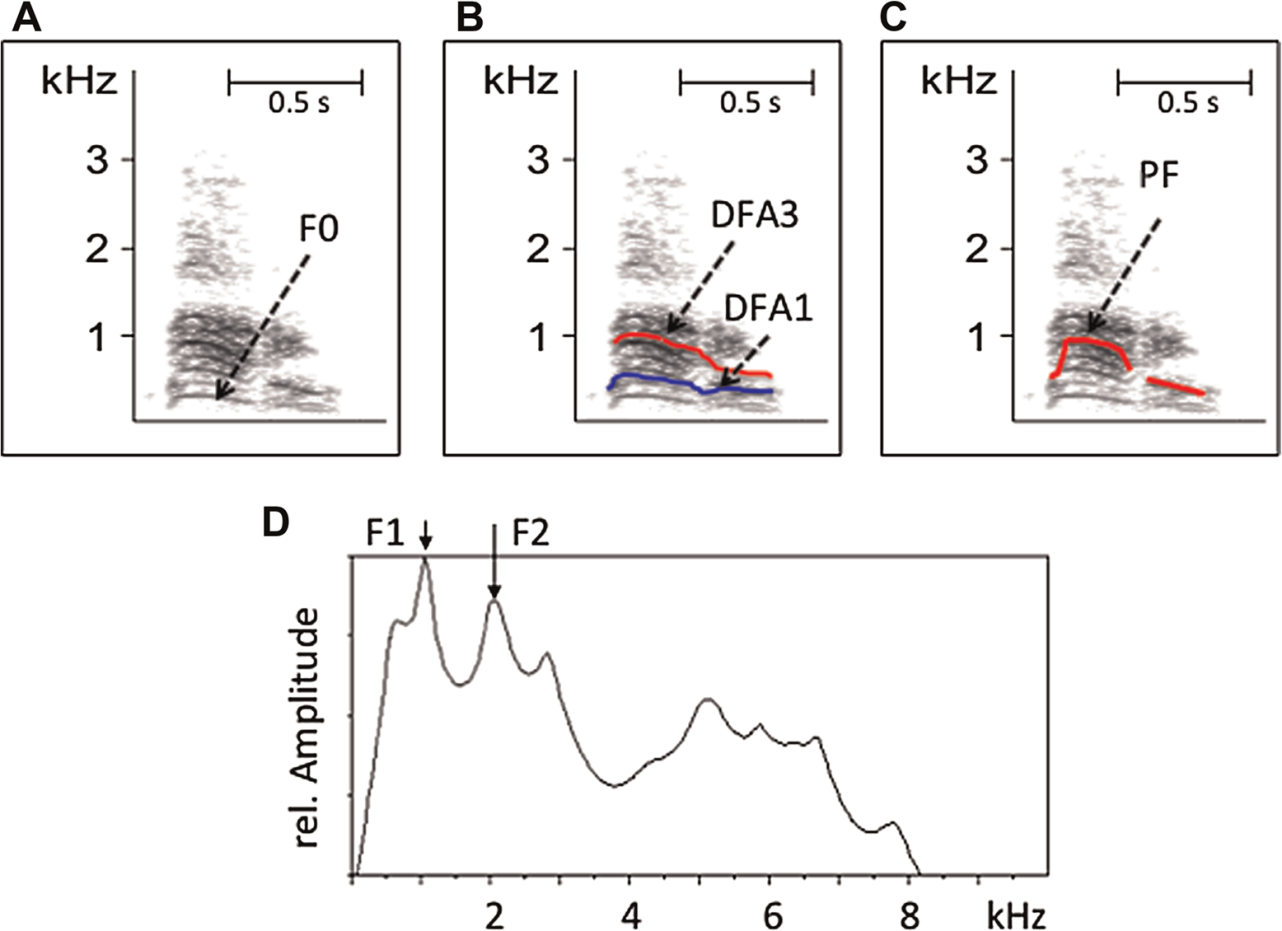
Estimation of different acoustic parameters from a male baboon clear bark. A: Fundamental frequency (F0). B: First and third quartile of the distribution of frequency amplitudes (DFA1 and DFA3). C: Peak frequency (PF). D: First and second formant (F1 and F2). The y-axis in panel D depicts the relative amplitude, normalized to the maximum amplitude in the call.

In our own research, we largely relied on measurements of the actual energy distribution, without making any assumptions about the sound production mechanisms. The advantage is that such methods can be applied to a wide variety of different call types, ranging from noisy calls to high pitched whistles. The latter do not lend themselves to formant analyses, which for a reliable identification of the vocal tract filter function require a broad frequency spectrum, such as a noisy signal or a harmonic signal with many harmonics and ideally a constant F0. We found the following call characteristics generally informative, although their specific value may vary between species or research questions. The first concerns the tonality of the call, including the potential identification of the fundamental frequency. Automated extractions of the fundamental frequency are mainly based on an autocorrelation function, which is applied to the different time segments of the call. Such algorithms can also be used to classify the call or call segments. A call can be classified as noisy if the autocorrelation function shows no discernible amplitude peaks, and as complex if some peaks can be detected, which may, however, not be periodic. If regularly spaced (periodic) peaks can be detected, this justifies the classification of the call as tonal and allows for the automated extraction of the fundamental frequency (Fig. 2A). In addition to the mean value, other features such as the start, minimum, or maximum value, or the modulation of the fundamental frequency may be determined. Sometimes, an algorithm that is used to identify the fundamental frequency may erroneously detect the first harmonic or F0/2. This is because the algorithm to estimate the F0 requires a sufficient number of harmonics for reliable identification. High-pitched sounds like screams may have less than three harmonics, which is not sufficient to reliably derive the F0. Another cause of unreliable results may be that due to the filter function of the vocal tract only few harmonics reach a certain threshold. In all such cases, it is difficult to make a reliable automatic F0 calculation, but the human eye easily spots such errors. Under such circumstances, it is advisable to restrict the search space for the algorithm to yield reliable results, and to monitor the quality of the results by eyeballing the values. Numerous nonhuman primate calls exhibit diagnostics of period-doubling bifurcations (insertions of subharmonic episodes with approximately F0/2, 3F0/2, etc.; see [Wilden et al., 1998] for details), indicating that non-linear effects play a role in their production [Fitch et al., 2002]. It is important to closely inspect spectrograms for such signs, because they may affect the identification of the fundamental frequency when automated procedures are used. Again, we recommend visually guided analyses with the aid of a harmonic cursor to deal with such sounds.

Another set of variables that has proven highly informative is the statistical distribution of the frequency amplitude in the spectrum. The first step consists in calculating the overall energy in each time segment. Subsequently, one can determine the frequency at which the distribution of the amplitude in the frequency spectrum (hereafter “distribution of frequency amplitudes”) reaches the first, second, and third quartile of the total distribution, respectively (Fig. 2B). Specifically for noisy calls, these variables may reveal meaningful variation. Again, different aspects of these variables such as the mean, median, start value, etc., can be extracted.

Third, we also often consider the so-called dominant frequency bands (dfb). Amplitudes that exceed a given threshold in a consecutive number of frequency bins characterize these dominant frequency bins (Fig. 2C). In tonal calls, the first dominant frequency band is usually equivalent to the fundamental frequency. Such frequency bands can be detected using different thresholds, so that more or less fine-grained differences in the energy distribution can be detected. Further important variables are the frequency and the modulation of the peak frequency, the frequency with the highest amplitude in a certain time segment, as well as the mean and maximum frequency range.

#### Formant analysis

The main alternative to the extraction of variables from the sound spectrogram is the application of LPC analysis to characterize the filter-related variation in the frequency spectrum (Fig. 2D). A critical step in LPC analysis is the setting of the number of coefficients in the analysis, which is decisive for the number of peaks that can be detected. For some analyses, the number of coefficients can be kept constant; for others, researchers may want to vary the number in relation to the call characteristics, using the spectrogram for visual aid. In such cases, it is also essential to determine the observer reliability. Depending on the characteristics of the call, it may finally be necessary to apply a pre-emphasis filter (e.g., 6dB/octave), in order to increase the higher frequency components.

As explained in detail elsewhere [Fitch & Hauser, 2003], the filter characteristics of the voca1l tract depend on the length and the shape of the vocal tract. Although mammalian vocal tracts do not constitute straight tubes [Fant, 1960], a number of more recent papers made this assumption as a simplified approximation. Thus, on the specific assumption that the vocal tract constitutes a straight tube that is closed on one end and open at the other, the spacing between the subsequent formants should be even. The longer the vocal tract, the more closely spaced are the formants. The average distance between formants has become known as “formant dispersion” and has been used as an indirect proxy to body size or vocal tract length. Fitch [1997] calculated formant dispersion applying Equation (1):


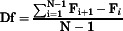
(1)

where Df is the formant dispersion (in Hz), N is the total number of formants measured, and F_i_ is the frequency (in Hz) of formant i.

An alternative method is to plot the (mean) values of the formant peaks against (2_i_ − 1)/2 increments of the formant. Reby and McComb [2003] then calculated the formant dispersion by fitting a linear regression line to the observed values, using an intercept equal to 0. One can then “reverse engineer” the actual vocal tract length by applying Equation (2):


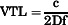
(2)

**Fig. 3:**
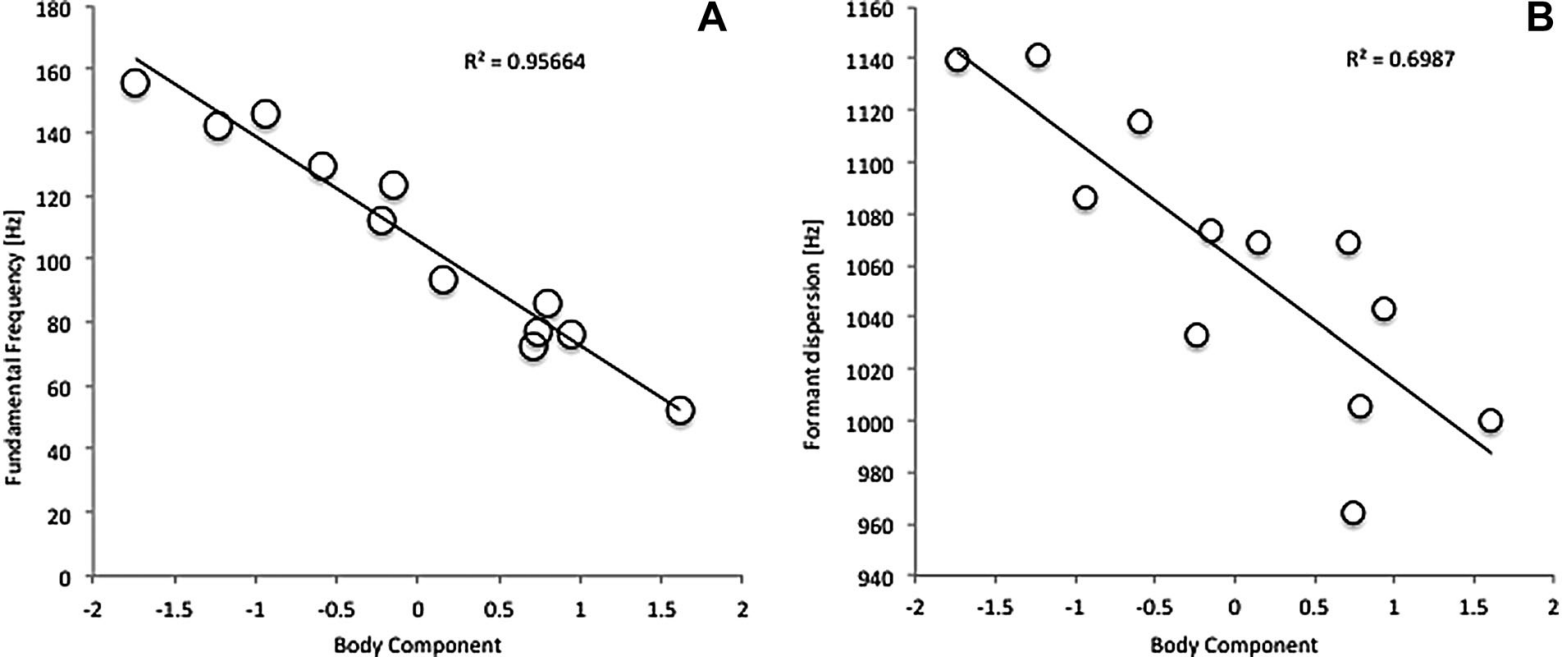
Relationship between acoustic characteristics of grunts and body size in hamadryas baboons. A: Fundamental frequency versus body size; B: formant dispersion versus body size. Redrawn from Pfefferle and Fischer [2006], with permission from Elsevier. Formant dispersion was recalculated according to Reby and McComb [2003].

Fitch [1997] found a high correlation between the actual VTL as measured by radioscintigraphy (r = 0.915 with oral VTL and 0.852 with the nasal VTL). Hamadryas baboon grunts, in contrast, revealed a tight correlation between fundamental frequency and body size (Fig. 3A), while the correlation between body size and formant dispersion was less strong (Fig. 3B) [Pfefferle & Fischer, 2006].

There is some danger of circular reasoning with LPC analyses, as the number of coefficients is derived from (theoretical) assumptions of the vocal tract length. These in turn determine the number of formants, and formant spacing, which is then sometimes used to infer VTL. Setting rather arbitrary cut-offs for the frequency range may also constitute a problem. Another, more general issue, is detecting the formant peaks, either because they are hardly discernible, or because there may be double peaks. In such cases, it is essential to stick to clear decision criteria to yield reproducible results. But even if strict criteria are applied, the number and location of formants may vary between different call exemplars recorded from the same subject.

#### Automated feature extraction and quality control

Measurements from spectrograms can also be made by hand using a cursor, which is often the only option when the signal-to-noise ratio is low, or when insect or bird sounds interfere with the signal. If the signal is relatively “clean,” software programs can be used to automatically extract different call features. In our experience, user guided automated measurements yield the most reliable results. That is, the program is used to extract the features, and the human observer confirms the accuracy of the measurements through visual assessment. Likewise, results from formant analyses need to be checked, for example, by comparing the identified peaks to the corresponding spectrogram. Widely available software programs as well as custom programs such as LMA (developed by Kurt Hammerschmidt) offer different routines that can be used in different combinations. In any case, it is essential to assure the quality of the measurements by plotting the data, and by checking the distribution of measurements, and their general plausibility. Thus, while automated measurements greatly speed up the collection of data, much of that “saved time” needs to be invested in quality assurance.

### Statistical Analyses

A thorough discussion of the various aspects of the statistical analyses that are possible with acoustic data is beyond the scope of this paper. Analyses may include simple correlations [Fitch, 1997], linear models [Fischer et al., 2004], analyses of variance [Owren & Bernacki, 1988; Owren et al., 1997], etc. In recent years, generalized linear mixed models, which allow the incorporation of subject ID as a random factor, have become commonplace to test hypotheses formulated at the outset of the study [Fischer, 2004; Pfefferle et al., 2008a]. Alternatively, if one is pursuing a more exploratory approach, classification techniques such as discriminant function analysis are useful [Fischer et al., 2001a]. If a large number of variables are entered in multivariate statistical analyses, problems with the multicollinearity may arise. Therefore, it is necessary to screen the variables for possible correlations, and to either exclude correlating variables, or to use principle component analyses to group acoustic features into orthogonal, uncorrelated factors. The danger here is that informative variation may be lost, however.

Discriminant function analysis (DFA) has proven to be a very powerful tool. DFAs establish a number of so-called discriminant functions to optimally distinguish between different groups, such as calls given in different contexts, or uttered by different individuals. Akin to regression analysis, the DFA generates a list of those variables with the highest explanatory value (i.e., the highest correlation with the discriminant functions). In addition, the DFA provides a classification procedure that assigns calls on the basis of their acoustic characteristics to one of the groups that were established by the discriminant functions. It is important to understand that the DFA is primarily an exploratory tool to generate hypotheses, for instance about which parameters are decisive. It is thus not permissible to use the same dataset to establish the discriminant functions, and to test the results of the classification procedure statistically. In the ideal world, one would have two entirely independent datasets, one to generate the functions, and another to classify the calls. This is rarely the case, specifically, when working with calls recorded from wild animals whose calls are difficult to record in the first place. Therefore, one may split a dataset in half if it is large enough, use a “leave-one-out” classification procedure, or apply bootstrapping methods. Further pitfalls with discriminant function analysis are discussed in detail elsewhere [Mundry & Sommer, 2007]. Importantly, the use of the classification procedure to assess the assignment to different groups (e.g., contexts or individuals) habitually yields overestimations [Mundry & Sommer, 2007]. Thus, the results have to be treated with care (and skepticism). Irrespective of these shortcomings, we used discriminant function analysis successfully to select calls of differing degree of similarity for subsequent playback experiments [Fischer, 1998; Fischer et al., 2001b]. Specifically, using calls from just two contexts, we assessed the so-called discriminant score, which is assigned to each call exemplar, to identify calls with either highly diverging or rather similar discriminant scores for tests in habituation-recovery experiments (see below). Furthermore, the DFA calculates the “assignment probability,” a value between 0 and 1 reflecting the certainty with which a call can be attributed to a specific category. There is a nonlinear relationship between the discriminant score and the assignment probability.

Studies of nonhuman primates are frequently hampered by a small number of subjects. One should be aware of the limitations before embarking on a cumbersome study of (in an extreme case) one male and three females, in the search of sex-differences in call characteristics, for instance. In such cases, one has to make do with a simple description. To obtain a good idea of how many subjects are required for reliable statistical inference we advise estimating the power of the test (before beginning the study). There are freely available resources on the web (e.g., GPower), which facilitate these calculations. One possibility is to determine a priori the needed number of subjects, calculated as a function of the power level 1-β, the significance level α, and the to-be-detected population effect size. If the study is already completed or the number of individuals is fixed, it is important to calculate the test sensitivity (population effect size is computed as a function of α, 1-β, and N) [Cunningham & McCrum-Gardner, 2007; Faul et al., 2007].

Generally, the statistical analysis will generate a set of research questions regarding the classification of the calls by the animals themselves. That is, acoustic analyses frequently form a formidable basis for planning the next field trip, to conduct playback experiments. Of course, as we will explain below, there are numerous other questions that can be addressed using field playback experiments.

Overall, the key issue in acoustic analysis is finding the best approach and the right settings to extract features with sufficient confidence. If the recordings are of good quality, automated extractions are preferable, because they yield fully replicable results. Nevertheless, the output of automated procedures needs to be validated by careful inspection. Whenever subjective assessments are made, the observer reliability needs to be ensured.

The web provides a variety of information about sound analysis. Webpages of software companies and research institutes provide valuable resources on general or specific topics about sound recording and sound analysis. In this way one may find specific tools or software code, like tool boxes to estimate formant structure with PRAAT or MATLAB code for certain aspects of sound analysis. Many of these packages are free for scientific use.

## PLAYBACK EXPERIMENTS

### General Aspects

In this section, we outline some factors that we consider crucial for a playback experiment to succeed, and illustrate our points by a review of study designs. The selection of studies is somewhat eclectic, with a bias towards our own first-hand experience when it comes to the things that can go wrong (there are many). We only discuss papers that have been performed on wild or semi-wild populations, and we largely limit ourselves to acoustic playback experiments. There are a number of other field experimental techniques [reviewed in Zuberbühler & Wittig, 2011], such as the presentation of stuffed predator models [Arnold et al., 2008] or photographs [Schell et al., 2011].

#### Getting ready

All good playback experiments start with high-quality sound recordings (see above). Thus, before using calls, it is advisable to upload, inspect, and edit them using appropriate software. Calls should not show signs of overload, nor disturbances such as bird song or excessive insect noise. The desired calls are then extracted from the recording and saved as a separate file. Often, the sound before and after the desired stimulus is completely silenced, but the amplitude onset should not be too sudden (depending on the dynamics of the playback device). At this stage, temporal aspects such as bout duration or the inter-call interval may be manipulated. The amplitude of the different calls should be normalized, but this does not always translate into equivalent sound pressure levels (SPL) after broadcasting. Therefore, careful control of the SPL at the distance that is usually used in the experiments is essential. We discourage the filtering of the playback stimulus itself to eliminate background noise, as this often has unwanted side effects on the structure of the calls. Depending on the equipment used for playback, the files are then dubbed back onto tape, or uploaded on a solid-state recorder with which the playbacks are then conducted.

Clearly, field experiments lack the precision of their laboratory counterparts. It is simply impossible to control for all of the variation in the social and physical environment in natural habitats. A sufficient sample size and care with the design of the study is therefore important, assuming that possible confounds are leveled out across trials [Cheney & Seyfarth, 2007]. Because field experiments usually focus on natural, untrained responses to calls from conspecifics, predators, or other species, they can only reveal what animals naturally do or know, while they are not suited to explore perceptual limits. In other words, field playback experiments tap into the “just-meaningful-difference” rather than the “just-noticeable-difference” [Nelson, 1988; Nelson et al., 1990]. Their tremendous power stems from the fact that experiments with animals that live in a natural social and physical environment shed light onto the things that actually matter to the animals. Their responses to playback experiments are therefore likely to reflect natural, biologically relevant behaviors. These provide unique insights into evolutionarily important aspects of the animals' communication and knowledge about their environment.

The choice of the equipment depends on the necessity of obtaining high-quality recordings as well as the available budget. The good old Nagra Kudelski DSM Speaker Monitor (Nagra Audio, Cheseaux, Switzerland) unfortunately is no longer available, and those labs that still have an exemplar or two will likely guard it carefully. The “Nagra,” as it is fondly termed, is heavy and sturdy, with giant platines inside, but the sound quality is excellent. We have also worked with the BOSE Roommate II (Bose, Friedrichsdorf, Germany), which was good for playing back baboon grunts and barks, but again, this speaker is no longer available. In our lab, we have now added the DAVIDactive (Visonic, Berlin, Germany) system fitted with custom-made battery packs, which provides very good sound quality. Other labs have custom-built speakers. If the calls have low frequencies, and the amplitude of playbacks is high, larger speakers are necessary. Kitchen et al. [2003b], for instance, used an Electro-Voice SX-2000 loudspeaker (Burnsville, MN, USA) powered by a Pioneer GM-X922 amplifier (Pioneer, Tokyo, Japan) to broadcast male baboon loud calls. This piece was so heavy that it needed to be hauled around in a car or dug-out canoe. In any case, it is essential to choose a system that is sufficiently sturdy. Cables and jacks should fit well, and always have replacement cables at hand; they have a tendency to come apart in remote places. Use cables that do not get tangled up. A long cable (>10 m) allows the operator of the playback device to stay at some distance from the location of the speaker. As playback devices, we are presently happy with solid-state recorders that allow for saving the different sounds as different files, which can be selected shortly before an experimental trial, according to the situation encountered. This is much more accurate and faster than the early days' need to fast forward to a specific counter number using analogue tapes, or using little paper clips on reel-to-reel audio recorders [Seyfarth et al., 1980a].

The quality of the playback stimuli needs to be controlled by conducting trials out of earshot of the subjects, preferably far away from the study location. This also helps to become familiar with the setting up of the experiment and the playback devices. Gerhardt [1992] suggested to check the quality of the playbacks based on measurements of the test signals at the distance at which the subjects are typically receiving them to control the fidelity of sound reproduction. Some studies indeed provide qualitative and/or quantitative descriptions of the stimulus [Zuberbühler, 2000c], in addition to giving detailed information on sound pressure level at a given distance [Fischer, 2004]. Maciej et al. [2011] systematically assessed the degradation of different baboon call types in different habitats, played back at different heights, and re-recorded at different distances. Due to the enormous number of trials that can arise from such a design, however, most scholars will have to make do with checking the quality of the typical playback situation.

#### Setting up the experimental situation

**Fig. 4:**
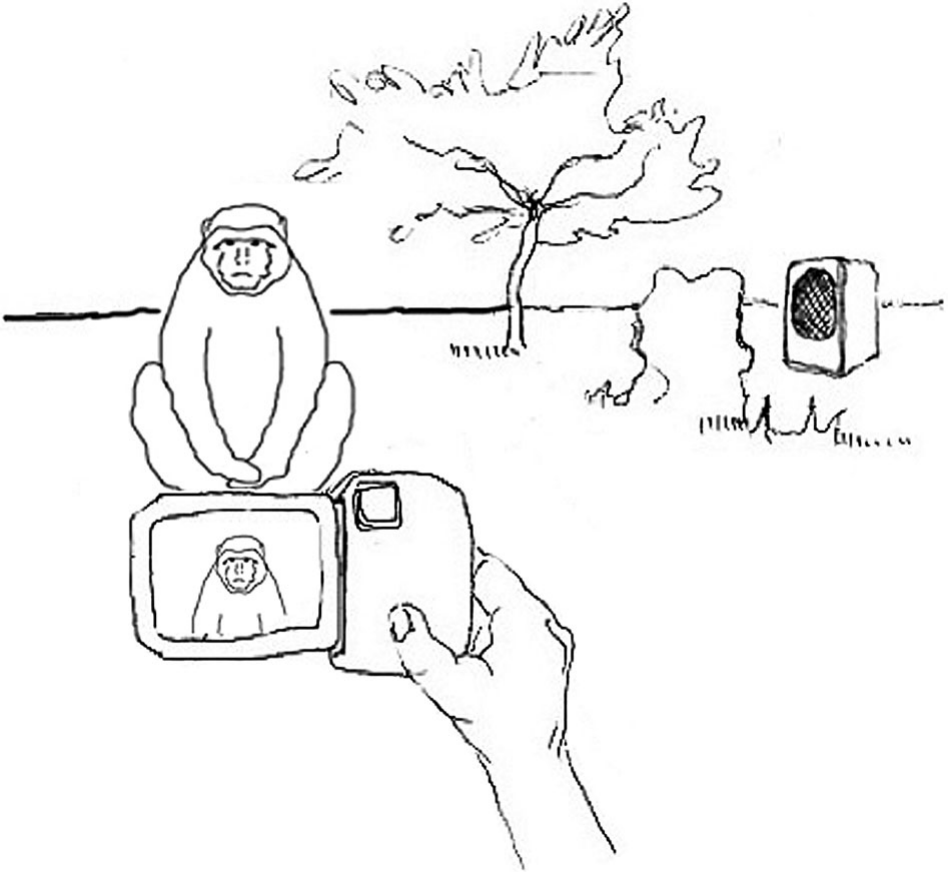
General set-up of a playback experiment. Ideally, the loudspeaker is set up at an angle of 90° to the subject, which is facing the video camera. The speaker needs to be hidden and the direction from which the call will be broadcast should be plausible.

Back in the field, the biggest challenge is the search for suitable situations that meet the criteria set up beforehand. Depending on these criteria, one may spend hours, if not days, until a playback trial can be conducted. Typically, two researchers work together, one is responsible for hiding the loudspeaker and operating the playback device, while the other ensures that all criteria are met and records the immediate behavior of the subject(s) using a video camera or sound recorder (Fig. 4). In our experience, it is a good idea to communicate using radios (walkie-talkies) with earphones. This also allows communication when visibility is poor. However, keep in mind that radios may fail when the signal is blocked in rugged areas. In these cases, consider establishing an additional antenna. Mobile phones may be a suitable means of communication in less remote areas. With nonhuman primates, utmost care is needed to create a “believable” situation in which the call is delivered. The loudspeaker(s) must be hidden well in tall grass, or behind bushes or trees. Once the animals understand that there is an artificial sound source, they rapidly cease to respond—unlike meerkats, *Suricata suricatta*, that repeatedly bring food to a loudspeaker wrapped in a plastic bag from which pup begging calls are emitted [Manser & Avey, 2000].

Once a suitable contextual situation is identified, one needs to ensure that the animal whose calls are to be broadcast is out of sight. Also, the loudspeaker(s) need to be placed in a “plausible” or at least in a “possible” location, that is, the direction of the individual whose calls are played should match the direction of the loudspeaker. This may mean that the loudspeaker needs to be hauled up in a tree. In any case, it should not be put directly on the ground, as this may lead to sound distortion. A small tripod or a backpack device with a metal rig is useful here.

Ideally, trials are conducted in an experimenter-blind design, in order to avoid unconscious selection of specific situations that make the presence or absence of a response more or less likely [Fischer, 2004]. In reality, this is not always possible, particularly, if several contingencies have to be met, such as specific previous interactions between individuals [Cheney et al., 2010]. The contingencies may vary considerably in their complexity. For simple alarm call playbacks, the researchers need to make sure that, to the best of one's knowledge, no predator is around. Often, such experiments are conducted after relatively long periods of calm, thus biasing against one's predictions. In other cases, a number of conditions need to be met at the same time, before an experimental trial can take place. For instance, in their study on kin-mediated reconciliation, Wittig et al. [2007b] waited for a fight to take place between two females. An experimental trial then took place when (1) the two females had separated for at least 5 min without interacting or vocalizing, (2) they were out of sight of each other, (3) the subject was out of sight of any member of her opponent's matriline, (4) the loudspeaker could be hidden at a distance of 5–8 m from the subject in the direction in which the aggressor had disappeared, (5) the subject was sitting or standing, and (6) not interacting with another group member. Given that the lists of conditions are often extensive, long data-collection periods are the norm.

It is important to consider the rate of delivery and the order of conditions. Before an experiment is started, it is essential to take data on the natural rate of occurrence of the calls to be used. One should aim to stay within the natural variation, and avoid a substantial increase in rate of occurrence. For alarm calls, this may mean long stretches between consecutive trials, while some frequently occurring social calls like baboon grunts [Cheney & Seyfarth, 1997] can be presented at a higher rate. Most experiments consist of one test condition and one or more control conditions. It is essential to carefully balance the order of presentation, as the response strength may fade with the number of trials and then constitute a confound. If possible, the side from which the call is presented, should also be varied systematically, and at the very least noted. GPS recordings of the loudspeaker locations relative to the focal animals allow to control post hoc for unwanted spatial biases.

Although playback experiments in group-living species usually measure the response of a single subject at a time, an unknown number of group members are likely to perceive the playback stimulus as well. Their responses usually remain unknown, and it is often unclear whether and to what degree the subject's response is affected by the response of others. Lemasson et al. [2008] explicitly addressed this issue by performing playbacks only when subjects were separated from other group members by ≥50 m in a habitat of high visibility. We repeatedly experienced that responses towards the playback stimuli decreased with an increasing number of trials, as subjects apparently learnt that they were not predicting anything of importance, sometimes after one playback trial only (C. Teufel and J. Fischer, unpublished data). Keeping the playback rate low, and maximizing the time between consecutive trials with the same subject are important precautions. Limits are imposed when the experiments require certain social configurations. In our experience, so-called habituation experiments are particularly problematic and should only be used on free-ranging animals when there is absolutely no alternative.

#### Recording responses

In the majority of playback studies, the responses are filmed on video, but depending on the dependent variable of interest, sound recordings, behavioral observations, or a combination may be chosen. When the behavior of a single subject needs to be captured, the experimenter with the camera will aim to place herself in front of the animal at a good distance, while the speaker is hidden to the left or right back of the animal. In such a configuration, a head-turning response can be unequivocally determined. If the speaker is set up directly behind the animal, it can be difficult to identify the end of the orienting response. In practice, subjects frequently move around and will inevitably turn away from the ideal position. One should establish clear abort criteria for such cases. Aborted trials can serve as so-called “mock trials,” in which everything is set up but no sound is played. This helps to avoid cueing the animals. If one is lucky and never needs to abort a trial, such mock trials should be conducted on a regular basis.

If possible, video cameras with flexible screens should be chosen. This allows filming the subject while looking down at the screen rather than directly looking at the subject. Wearing a baseball cap that hides the researcher's eyes to some extent may help to prevent staring at the subject for a longer period of time. Typically, filming starts before the calls are broadcast so that baseline behavior can be taken into account. In some experiments, entire subgroups of animals [Cheney & Seyfarth, 1980] have been filmed. Following the playbacks, a number of studies additionally took data on the subject's behavior for a given time, for instance, for half an hour after the stimulus was broadcast. Specifically for “social” experiments, such data are invaluable [Pfefferle et al., 2008b; Wittig et al., 2007b]. These observations follow the standard rules for behavioral observations.

We advise researchers to record as much background information as possible in conjunction with each experimental trial and its specific situation, such as date, time of day, identity of subject, distance between subject and speaker, distance and location of the speaker relative to the subject, the playback file used, the general context, etc. This can be done in the form of a spoken comment directly onto the video film. According to our experience, it is a good idea to do this twice, once before the call is set off and once again at the end of the film. Because playback trials are often highly stressful events, this redundancy makes sure that all the essential information is available during data analysis. In addition, we take written notes, draw a sketch and take GPS positions of the set-up of each trial. We found it particularly useful to enter information about the experimental situation to a handheld computer, preferably in a form where all fields need to be filled out before one can proceed.

#### Analysis of the responses

If the behavior was recorded on videotape, film-editing software is necessary to assess the type and duration of response. Some consumer products can serve the purpose to measure response duration on a frame-by-frame basis, while high-end professional software such as Adobe Premiere (Adobe Systems, Inc.) offers more power in setting flags and screening the recording, at the expense of taking more time to become acquainted with the program. The coder should be blind to experimental condition, with the sound turned off. The onset of the call is normally visible in the audio track. A second independent coder should code ∼20% of the film clips, to determine the inter-observer reliability.

One important measure in many studies is the looking time duration, also known as the duration of the orienting response. There are in principle two drivers of looking time: importance and novelty, which may work in opposite directions. On the one hand, animals are expected to look longer towards stimuli that matter than to those that do not matter, for example the vocalizations of their kin compared to unrelated animals. At the same time, they should attend longer to novel than to familiar stimuli. In this regard, the orienting response can also be understood as an expression of surprise. Indeed, both humans and animals generally look longer towards unexpected stimuli than towards expected ones [Cheney & Seyfarth, 1990; Fantz, 1965]. The predictions for the looking time duration thus depend on the details of the experimental design and the research question. In addition to or in lieu of measuring the looking time duration, the number of looks can be recorded [Crockford et al., 2007].

Assessing the looking time duration is not always trivial. In our experience, the onset of the response (in case of a head-turn) can often be well determined as at least Barbary macaques and baboons briefly close their eyelids before turning the head. The offset of the response is more difficult to determine. Specifically if the animals start to “gaze into the distance,” it is sometimes not clear whether this should still be viewed as “looking at the loudspeaker.” As far as possible, consistent criteria need to be developed and applied. We also often found that animals first orient to the speaker, then look around, and then resume looking at the speaker [e.g., Pfefferle et al., 2008b]. In such situations, we separately determined the duration of the first look and the total time spent looking at the speaker.

Another important variable is the latency to respond [Engh et al., 2006], which is considered to be a measure of the arousal level or the motivation to attend to the stimulus [Palombit et al., 1997]. In addition, a whole suite of measures can be taken, such as the distance walked, and the nature, number, or duration of a vocal response, for instance. If these variables correlate, McGregor [1992] recommended using a principal components analysis to generate single composite measures that are statistically independent from each other, which can then be used for statistical testing.

In other cases, responses can be categorically different. For instance, Seyfarth et al. [1980a] scored whether the animals “ran into trees,” “looked on the ground,” or “looked up” in response to playbacks of vervet monkey alarm calls. Other studies noted whether the animals approached [e.g., Palombit et al., 1997; Pfefferle et al., 2008b] or retreated from the speaker [e.g., Engh et al., 2006], or whether they produced vocal responses [e.g., Zuberbühler, 2000a]. While the statistical analysis of simple binary responses is straightforward, the assessment of categorically exclusive responses is to date not possible with mixed models (R. Mundry, personal communication). In addition to the immediate behavior, longer term responses may also be considered. These include variation in social behavior, such as interaction rates, the quality of interactions or identity of partners following during a predetermined time period (e.g., 30 min) after the presentation of the stimulus [Engh et al., 2006; Wittig et al., 2007b]. A summary of the various response measures is given in Table 2.

**Table II:** Common Behavioral Responses Measured in Playback Studies

Response	Description	References^a^
Looks		
Occurrence	Absence or presence of visual orientation towards loudspeaker	Cheney and Seyfarth [1980]
Duration	Duration of visual orientation towards loudspeaker	Bergman et al. [2003]
	Duration of first look	Pfefferle et al. [2008b]
Bouts	Frequency of subsequent bouts of visually orienting towards speaker	Crockford et al. [2007]
Vocalizations		
Occurrence	Whether or not certain calls occur	Zuberbühler [2000b]
Frequency	Frequency of calls of a given type	Bshary [2001]
Movements		
Occurrence	Whether or not subjects: move (e.g., at least one step)	Pfefferle et al. [2008b]
Direction	Approach loudspeakers	Crockford et al. [2007]
	Move away from playback area	Engh et al. [2006]
	Move to a specific substrate (e.g., run into trees, into cover)	Cheney and Seyfarth [1980]
	Compass direction of movement from onset to offset	Bshary and Noe [1997]
Duration	Time spent traveling	Bshary and Noe [1997]
Distance	Distance traveled between onset and end of movement	Bshary and Noe [1997]
Latency	Time between onset of playback call and onset of movement	Kitchen et al. [2003a]
Social interactions		
Occurrence	Whether or not certain social interactions occur	Cheney and Seyfarth [1997]
Initiator	Whether or not subjects initiated social interaction	Cheney and Seyfarth [1997]
Nature	Types of social interaction occurring after playback stimuli	Cheney and Seyfarth [1999]
Other behaviors		
Occurrence	Whether or not specific other behaviors occurred (e.g., bipedal standing)	Seyfarth et al. [1980a]
Latency	Time between playback stimuli and first occurrence of specific other behavior (e.g., tolerance of the opponent's proximity)	Wittig et al. [2007b]
General response		
Latency	Time between onset of playback call and onset of response	Fischer [2004]
Duration	Compound measure of several responses, for example time spent looking plus time spent traveling to loudspeakers	Fischer [2004]

a Although some responses are used in many studies, only a single reference is given.

A matter of debate is whether or not a specific baseline behavior should be established. Some authors explicitly state that the critical behavior (looking into the direction of the speaker) was not observed in the time period before the playback, and thus no baseline value needed to be taken into account [Fischer, 2004]. Other studies [Lemasson et al., 2008; Palombit et al., 1997] used the duration of orienting towards the loudspeaker in the 20 sec after the playback minus the duration of orienting in the same direction in the 20 sec before the playback. Somewhat problematically, this may lead to negative looking time durations.

#### Statistical issues

As a rule of thumb one should keep in mind that the statistical power depends on the number of subjects, not on the number of trials done with each subject. That is, if you test the same individual (or the same group) 20 times with one stimulus and 20 times with another, the sample size is 1, not 20 (in a repeated measures design). On the other hand, if you test 40 individuals with two stimuli, putatively representing two different call types, your statistical inference is limited to these two stimulus exemplars, not to the class you assume they represent. Therefore, a different stimulus in each playback trial should ideally be used to avoid that subjects simply respond to some unwanted, unusual property of a single call (“simple pseudo replication;” [Kroodsma et al., 2001; McGregor, 1992]). However, some authors of primate playback studies have argued that specific stimuli occur so seldom in nature that it is virtually impossible to obtain a sufficient number of exemplars. For example, only very few recordings of extremely rare leopard growls were available for studies examining the responses of monkeys towards signs of predators [Zuberbühler, 2000a]. This limited the possible inference [McGregor, 1992].

Technically, modern analytical techniques such as Generalized Linear Mixed Models allow the inclusion of subject and stimulus identity as a random factor in statistical analyses. However, the number of factors (k) that can be meaningfully tested depends on the sample size (n). There are no strict rules, but recommendations range between [n ≥ 6 × k] to [n ≥ 15 × k] (R. Mundry, personal communication). Therefore, the best way out is to maximize the number of stimuli used—that is use each stimulus only once, or balance their usage. For instance, in an experiment tapping into recognition of the maternal voice, Fischer [2004] used the same stimulus as “maternal scream” for one infant and as “non-mother” scream for another infant in the same group. Such balanced designs allow controlling for the possibility that all maternal screams sound different from non-maternal screams, for instance.

In any case, it is important to bear in mind the limitations of the statistical inference that are possible with a given design. Because studies typically take many months, if not years, to complete, even the best design can be torpedoed by predation events, emigrations, changes in rank, etc. The problem may be exacerbated when dyads are used in the experiments. For instance, Engh et al. [2006] investigated whether subordinate females' responses to threat grunts of higher ranking females depended on the nature of the previous interaction. Not all of the 22 females could be included in the experiment, and not all of those that were used could be tested in all conditions, due to hierarchy effects, deaths and the fact that some females never interacted. As a result, the authors conducted 21 trials involving 12 dominants and 10 subordinates, and used each subject repeatedly in the same combination in different conditions, and also in different combinations, so that a specific female could be the dominant partner to two different subordinates. The authors thus decided to analyze their data twice, once by pooling their data, and once by averaging the responses of subjects, although strictly speaking, this “double testing” is not correct [Engh et al., 2006].

### Experimental Designs and Research Questions

Experimental designs can vary widely in terms of their complexity, ranging from simple presentations of a single call from one speaker, to repeated presentations at flexible intervals, to complicated variation of contextual and acoustic information. The single-speaker design is technically the simplest case, where only one speaker is set up, from which either a single call, a call bout, or even a combination of calls recorded from different animals can be played. The use of two different speakers delivering different calls from different locations is also possible, and not surprisingly, much more difficult to execute, especially when the exact timing of events is crucial. Below, we broadly group the different experimental designs by research question, namely the investigation of (1) context- or caller-related acoustic differences, (2) the importance of the social relationship between the putative caller and the subject, including the social function of calls, and (3) social knowledge. Two special cases, the habituation-recovery design and the two-speaker choice test are discussed separately, as they cut across these broad categories.

### Acoustic Variation in Relation to Context or Quality of the Sender

The typical research question would be whether observed acoustic differences are salient to the listeners; the experiments thus consist of the presentation of acoustically distinct calls that were originally given in response to different stimuli, such as different predator categories, in different social contexts, or that reflect variation in caller condition.

Following the playback study on vervet monkey responses to conspecific alarm calls [Seyfarth et al., 1980a], a number of studies investigated whether other species show similarly distinct responses to variation in different alarm calls. For instance, Fichtel and Kappeler played back different calls of aerial and terrestrial predators to redfronted lemurs, *Eulemur fulvus rufus*, and to white sifakas, *Propithecus verreauxi verreauxi*. Both species responded with specific alarm calls only to the calls of aerial predators, whereas the calls given in response to calls from terrestrial predators also occurred in other contexts [Fichtel & Kappeler, 2002]. Similar studies were conducted in Barbary macaques [Fischer et al., 1995], tamarins [Kirchhof & Hammerschmidt, 2006] and forest guenons [Zuberbühler, 2000d, 2001].

In numerous species, the relevant acoustic variation does not appear to be the structure of single calls, but the composition of the entire call bout [Arnold & Zuberbühler, 2006, 2008; Schel et al., 2009]. To test whether listeners can extract this potential information, the playback consists of broadcasting natural or edited sequences of sounds. For instance, Arnold and Zuberbühler found that putty-nosed monkeys, *Cercopithecus nictitans*, give sequences of “pyows” and “hacks” in response to different aerial and terrestrial predators, or when they initiate a group movement. The composition of the sequences varies systematically in relation to the original context, and listeners respond differently to different types of sequences, thus supporting the view that listeners attach different meaning to different call combinations [Arnold & Zuberbühler, 2008].

Primate vocalizations may not only vary in relation to predators but also the quality of an interaction. For instance, the copulation calls of female Barbary macaques, *Macaca sylvanus*, differ acoustically in relation to cycle stage: calls at the beginning of the cycle are significantly shorter and have a lower mean dominant frequency than during peak swelling, when the ovulation is most likely to occur, and this variation is salient to male Barbary macaques, who respond more strongly to playbacks of calls recorded during peak swelling [Semple & McComb, 2000]. Note, however, that the calls do not clearly indicate the timing of ovulation [Pfefferle et al., 2008a]. Intriguingly, female Barbary macaque copulation calls given during copulation that resulted in ejaculations and those given during non-ejaculatory copulations differ acoustically. Playback experiments revealed that males looked significantly longer towards the loudspeaker after hearing ejaculatory calls than after hearing non-ejaculatory calls. Also, they spent more time walking and more time in close proximity to females after having heard ejaculatory calls [Pfefferle et al., 2008b].

Acoustic variation in relation to physiological state can also be found in the loud calls (“wahoos”) of male chacma baboons, *Papio ursinus*. These vary with the rank of the caller [Fischer et al., 2004]. When playback experiments systematically varied different features of the calls, listeners attended to variation in the two most prominent features that correlated with rank, namely the fundamental frequency and the length of the “hoo” syllable [Kitchen, D., Cheney, D.L., Engh, A., Fischer, J., Moscovice, L., Seyfarth, R.M., unpublished data]. Calls not only correlate with physiological but also with emotional state. For instance, chimpanzee, *Pan troglodytes schweinfurthii*, screams differ in relation to the role in a social conflict [Slocombe & Zuberbühler, 2005], and this variation appears to be perceptually salient [Slocombe et al., 2010].

The results of these experiments indicate that there are no fundamental differences in the experimental design and the assessment of the responses in relation to whether the calls “refer to” some external event such as the presence of a predator, or variation in arousal or internal state [Wheeler, 2010]. The implications for the mechanisms underlying the so-called “functionally referential signaling” are discussed elsewhere in more detail [Wheeler & Fischer, 2012], but it should be noted that the cognitive mechanisms supporting the responses to different alarm calls or different mating calls are probably the same.

In sum, there are a number of factors that can give rise to variation in calls, and depending on the hypothesis to be tested, the simple playback of a call or a call sequence can shed light on listeners' assessment and responses to these calls. The majority of the published data support the view that nonhuman primates are adept at distinguishing minor variation in calls, if this is related to ecologically or socially relevant variation in the environment or caller state.

#### Integration of contextual information

Subjects' responses may not only depend on the variation in the acoustic structure of the calls but also on other factors, such as variation in context [Fischer, 2013]. This has important repercussions for the identification of “call meaning,” which is typically inferred from the responses. An attempt to disentangle the relative contributions of variation in the signal and variation in context can be made by systematically pairing calls with different context. One early such attempt was made by Rendall et al. [1999], who played so-called “move” and “infant” grunts of baboons in situations, in which either actual move or infant grunts are typically heard. The responses clearly depended on both aspects. For instance, subjects were most likely to respond to the playback of move grunts by uttering move grunts themselves when the calls were presented at the edge of an “island,” where animals typically embark on crossings of the surrounding flood plains [Rendall et al., 1999]. Interestingly, Pfefferle et al. [2008b] found that male Barbary macaques only paid attention to variation in copulation call characteristics, while they ignored other potential sources of information about the receptivity of the female. Specifically, they presented copulation calls recorded during a time of high likelihood to conceive during the time of maximum swelling, and outside the females' sexual cycle. Male responses did not differ in relation to cycle stage.

#### Prime probe experiments

A more elaborate way to assess the integration of contextual and acoustic variation is to experimentally manipulate the context, for instance by presenting stuffed predator models before the playback, or, alternatively by first presenting some calls, and then later some others. This approach has become known as the “prime-probe” design. In a series of elegant experiments, Klaus Zuberbühler applied this method to explore the knowledge and communication of forest guenons [Zuberbühler, 2000b]. Among other aspects, he investigated whether male Diana monkeys, *Cercopithecus diana*, understand the semantic content of the alarm calls that chimpanzees give in the presence of leopards, *Panthera pardus*. These monkeys have two important predators, chimpanzees and leopards, and use two strategies to defend themselves against them: a conspicuous strategy in the presence of leopards (i.e. alarm calling), and a cryptic strategy in the presence of chimpanzees (i.e. silently vanishing). The conspicuous strategy tends to result in the leopards leaving the area upon being detected, whereas the cryptic strategy seems to result in the chimpanzees not being able to detect their prey. Chimpanzees themselves sometimes fall prey to leopards, and use a loud alarm call upon detection of a leopard in the area. This led to the question of whether Diana monkeys only took the leopard alarm calls of chimpanzees as signs of a chimpanzee being present, or whether they also understand that the chimpanzee signal contains the information that a leopard is present in the area. Zuberbühler [2000b] presented two playback stimuli, a prime and a probe, separated by 5 min of silence, to different Diana monkey groups. In the decisive test condition, males heard chimpanzee alarm calls followed by leopard growls. In this case, prime and probe stimuli were acoustically different but correlated with the same event, namely the presence of a leopard. About half of the males responded conspicuously towards the chimpanzee alarm calls, and then weakly towards the leopard growls, whereas the other half responded weakly towards the chimpanzee alarm calls, but then strongly towards the leopard growl [Zuberbühler, 2000b].

#### Variation in social relationships

Playback experiments that tap into the modulation of behavior in relation to the social relationship either address the effects of long-term relationships such as kinship or friendship, or may explore more short-term contingencies such as the effect of the last interaction before the experiment. In the first category, the context in which playbacks are conducted is typically held constant, while the nature of the relationship varies, whereas the reverse is true for the latter category. Regarding the importance of long-term relationships, rhesus monkeys, *Macaca mulatta*, for instance looked longer towards playbacks of their kin compared to other young group members [Rendall et al., 1996], while young Barbary macaques responded more strongly to playbacks of their mothers' calls [Fischer, 2004]. Palombit et al. [1997] investigated the function of baboon friendships. In one of their playback experiments, they examined whether the responses of males towards the distress calls of females varied systematically with their relationships to those females. For this purpose, female distress calls were played back to male friends and to non-friends (control) of similar social rank who had a friendship with another female. As expected, females' screams elicited significantly stronger responses from friends than from the control males [see also Lemasson et al., 2008].

Alternatively, the differences in the previous interaction between the caller and the recipient, or a relative of the caller and the recipient can be systematically exploited, as in the experiment by Engh et al. [2006], mentioned above. In another experiment, Wittig et al. examined whether vocal support in agonistic interactions function as vocal alliances. Female baboons heard the same female's threat-grunts after being threatened by a signaler's relative or an unrelated female. As a control, the threat grunts were played after an affiliative interaction with the signaler's kin. Subjects avoided both the signaler and her relatives in the condition mimicking vocal support by kin than in the control conditions [Wittig et al., 2007a]. Cheney et al. used a similar approach to assess the “contingent reciprocity” in chacma baboons. They played back threat grunts of a female baboon after she had previously engaged in grooming with the subject, or after she had threatened the subject. They found that subjects were more likely to approach the speaker after a previous positive compared to an agonistic interaction—presumably in support of the partner [Cheney et al., 2010].

#### Social knowledge

An experimental design using combinations of stimuli has been successfully used to investigate baboon knowledge of third-party relationships. The general (and ingenious) idea is to present not just one call or call series given by one individual, but instead to mock an interaction between two (or more) different animals, such as a threat vocalization followed by a fear bark (signaling submission). Since both realistic and unrealistic combinations of calls are used as playback stimulus, the paradigm opens the way to presenting stimuli that are violating the expectations of subjects [Hauser, 1998b].

One of the first such experiments was devised by Cheney et al. [1995], who played sequences of calls mimicking a social interaction. Specifically, they exploited the fact that dominant female baboons, when approaching subordinate females with infants, typically emit a series of grunts, to which mothers sometimes respond with submissive fear barks. In the experiments, the roles were now reversed: now a lower ranking female appeared to be grunting to a higher ranking female, who responded with fear barks. In the control condition, the test subjects heard the same sequence, but at the end, grunts from a third female that was higher ranking than the two other subjects were added, making the sequence of interactions plausible again. The analysis of looking time revealed that subjects responded more strongly to the implausible interaction than to the plausible one, indicating that they are aware of the hierarchical relationships in their social group.

Bergman et al. [2003] used a variant of this design to show that these baboons simultaneously classify group mates by social rank and by affiliation to a matriline, a form of higher order classification that up to that point had been viewed as uniquely human. The sequence of calls that they used mimicked a fight between two females, and consisted of a series of threat-grunts of one female, followed by the screams of the other. These baboons live in a society with a stringent and nested hierarchy where all members of a given matriline occupy adjacent ranks. Because threat/submission interactions are clearly unidirectional, some call combinations are very likely to occur in daily life while others are virtually non-existent. The study rested on the idea to broadcast call sequences mimicking highly unlikely female rank reversals, namely a lower-ranking female uttering threat calls to which a higher-ranking female responded with screaming. Subjects were tested in two test conditions, the first suggesting a rank reversal within a matriline, and the second a rank reversal between matrilines. In control trials, the sequence was consistent with the actual dominance hierarchy. Subjects responded most strongly to rank reversals that cut across matrilines, while there was no significant difference in the responses to rank reversals within matrilines or control trials. The experiments demonstrate that subjects are highly sensitive to putative upheavals that could potentially affect the entire female dominance structure in that baboon group, while they appear to care less about rank changes within matrilines [Bergman et al., 2003].

Crockford et al. [2007] investigated whether male chacma baboons pay attention to sexual consortships, that is, to highly transient relationships between a male and a receptive female that last up to several days. They placed two loudspeakers 40 m apart, with the test subject sitting calmly in the middle while feeding or resting. Each of the speakers broadcast a different call. In the test condition, subordinate males heard the consorting male's contact grunts from one speaker, and after a few seconds, the consorting female's copulation call from the other. This playback sequence simulated the fact that the consort pair had unexpectedly spatially separated, and that another male was now copulating with that female. Since a subordinate male possessing such information could gain a rare opportunity to mate opportunistically with the receptive female, strong responses to this scenario were expected. In the first control condition, the contact grunts of a non-consorting male were played from one speaker, and the receptive female's copulation calls from the other. This playback suggested that the consorting pair were busy copulating, while another male grunted 40 m apart. Therefore, only a weak response to this scenario was expected. The second control trial was conducted 24 hr after the actual consort had ended. Again, subjects heard the former consort male's grunts from one speaker, and the consort female's copulation call from the second. Thus, this control suggested that the consort pair had separated, and the female was now copulating with another male. Again, weak responses were expected to this scenario, since this scenario suggested what informed subjects already knew: that the consort had ended.

Indeed, males responded significantly more strongly to the test condition, compared to the control conditions. The number of looks towards the “female” loudspeaker was significantly higher during the test condition, compared to the control conditions, whereas the number of looks to the “male” loudspeaker did not vary across conditions. In the test condition, more approaches were scored to the speaker that had broadcasted the female copulation call, compared to the control conditions. These results suggested that male baboons routinely monitor other males' consortships, and Crockford et al. [2007] proposed that eavesdropping upon the temporal and spatial properties of male and female vocalizations uttered during consortship might be a strategy by which male baboons achieve sneaky matings.

#### Habituation-recovery paradigm

**Fig. 5:**
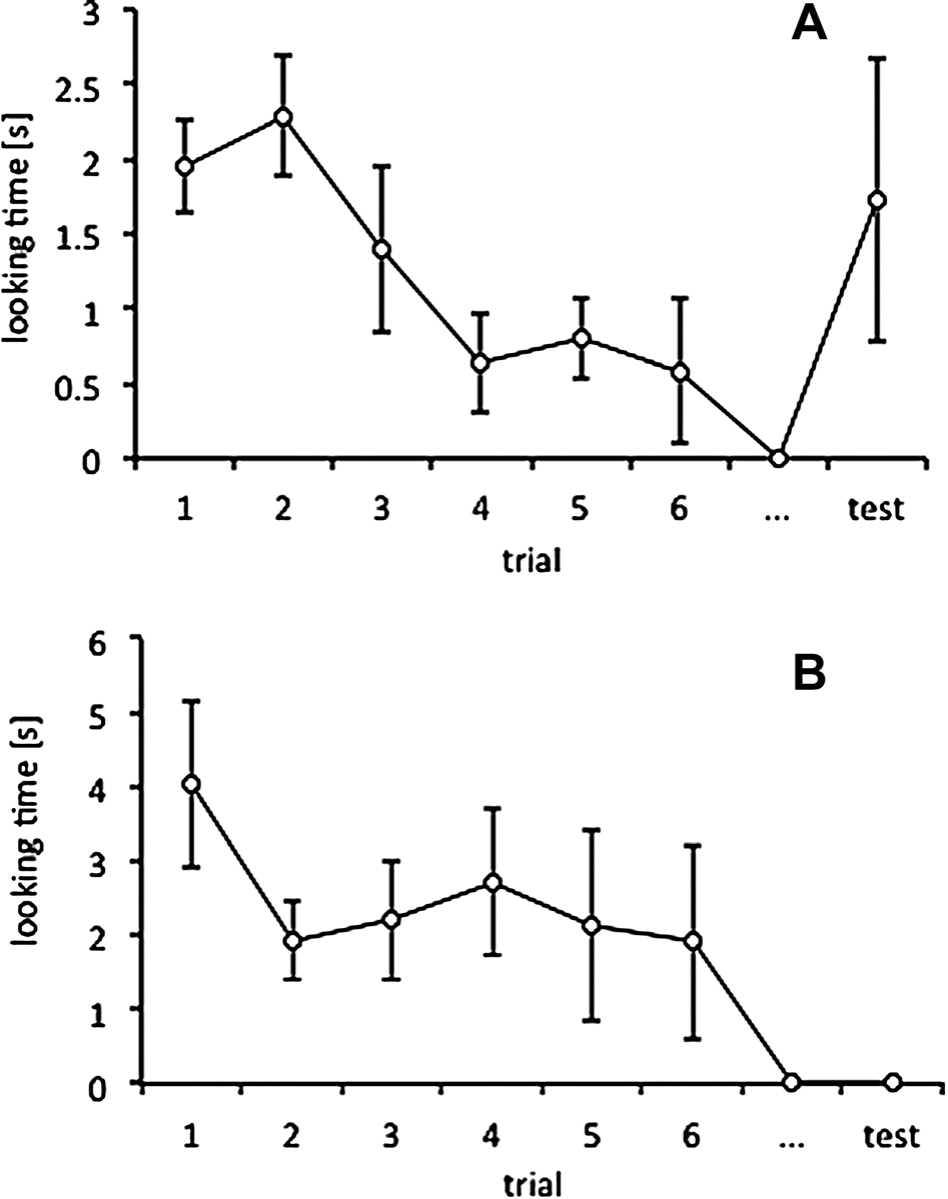
Time-course of looking time duration in a habituation-recovery experiment. A: Subjects were habituated with different Barbary macaque alarm call exemplars given in response to a human observer, and then tested with a call given in response to a dog after a variable number of habituation trials, depending on the behavior of the subject. After the subject had failed to respond in three trials, the test call was broadcast. The looking time shows a clear rebound of the animals' interest. B: As before, subjects were habituated with calls given in response to the human observer, and then tested with a novel call from the same category. The subjects showed no renewed interest. Data from Fischer [1998].

A paradigm that cuts across several of the research questions outlined above is the habituation-recovery paradigm, which is also known as “habituation-dishabituation” paradigm. The latter term, however, originally refers to a method to distinguish habituation from exhaustion at the neuronal level, and differs with regard to methodological details. We therefore prefer the term “habituation-recovery.” Because of its potential power but also the many problems associated with it, we discuss it separately. It has been used to study call categorization and the detection of unreliable signalers [Cheney & Seyfarth, 1988], the discrimination of different individuals within a group of kin [Rendall et al., 1996], the importance of acoustic similarity and “external reference” [Hauser, 1998a], and the discrimination of acoustically similar call types [Fischer, 1998; Fischer et al., 2001b]. The general idea is to establish one category by repeatedly presenting different call exemplars until the subjects begins to habituate (Fig. 5). The presentation ceases after either a predetermined number of calls or after the subject shows no discernible response in a number of presentations. Moreover, the spacing of the calls can be predetermined (fixed or jittered, with some variation in inter-call-interval) or interactive, where the next call is played when the subject turns its attention away from the calls. Finally, when the criterion has been reached, the test call, putatively belonging to a different category, is presented. Fischer [1998] tested how Barbary macaques responded to acoustic variation between two call types that form a graded continuum. In the tests, she presented calls that (i) stemmed from the same category, and revealed only minor acoustic variation, (ii) revealed the same amount of variation, but cut across the acoustic boundary, or (iii) a test call that was clearly acoustically distinct from the calls used for habituation. She found that animals did not show renewed responses if the novel call was from the same category, while they did respond both to small and large acoustic difference when they straddled the category boundary [Fischer, 1998]. Overall, the findings supported the view that the monkeys categorized these calls in a fashion similar to humans, when they categorize continuous acoustic variation in speech.

Although this approach is extremely powerful, a high number of trials needed to be aborted because the subjects moved away in the middle of the habituation sequence. Moreover, the repeated presentation of long and perhaps unnatural sequences of calls may lead to a general decline in response strength, as the subjects recognize the experimental situation as such. We made one further attempt to use this technique to explore the social knowledge of Barbary macaque females (C. Teufel and J. Fischer, unpublished data), only to find that most subjects hardly responded in the second or third trial, and that the overall response strength declined with an increasing number of trials. We therefore caution against the usage of this technique in the field. Clearly though, it can be highly useful in more controlled settings where the animals can be separated from their group and tested under confined conditions [Fitch & Hauser, 2004].

#### Two-speaker choice test

In the two-speaker choice paradigm, two stimuli are broadcasted from two spatially separated speakers simultaneously. This paradigm may work well to find out which signal out of two is more relevant than the other. It has successfully been used in birds [reviewed in Douglas & Mennill, 2010] and frogs [Gerhardt, 1994], and more recently also in deer [Wyman et al., 2011]. Hammerschmidt and Fischer [1998] used this approach to assess infant recognition by female Barbary macaques. Two speakers were set up in different directions, with the mother in the center, and screams of the mother's and another infant of the same social group were played simultaneously. Females looked longer towards the loudspeaker playing the screams of their own infants compared to the other. The advantage of such a design is that the subject is in the same motivational state—if the subject responds, the preference is usually unequivocal. Setting up the experiment is, however, difficult, and the question came up whether one could reliably determine what the subjects actually perceived.

In summary, acoustic playback experiments represent one of the most powerful types of field experiments. They can provide invaluable insights into the cognitive mechanisms that underlie primate communication and social behavior, and shed light onto the signals that are meaningful to the animals in an ecologically rich setting. However, experiments should not be done lightheartedly, but instead sparingly and only after collecting solid background data on the natural behavior of the species, which is a prerequisite for the solid interpretation of the results. Patience is crucial, as is the choice of the experimental design and the appropriate controls. If all these conditions are met, playback experiments are tremendously rewarding because they provide us with a glimpse into the animals' minds, and help us to understand the evolution of communication, intelligence, and social behavior.

## A FINAL REMARK

We hope that this paper will provide novices in the field with some initial guidance, and the more experienced researchers with a useful summary of current methods and approaches. The keys to success are careful planning, continuous quality control, and a great deal of frustration tolerance. If you make these part of your research endeavor, bioacoustic research does not only turn out to be fun but also yield deep insights into the communication of our closest living relatives.

## USEFUL RESOURCES

Bradbury JW, Vehrencamp SL. 2011. Principles of animal communication. Sunderland, MA: Sinauer Associates.

Brudzynski SM, editor. 2010. Handbook of mammalian vocalization. London: Academic Press.

Cornell Lab of Ornithology's Macaulay Library. Available online at: http://macaulaylibrary.org

Hopp SL, Owren MJ, Evans CS, editors. 1998. Animal acoustic communication: sound analysis and research methods. Berlin: Springer. p 1–421.

Setchell JM, Curtis DJ, editors. 2011. Field and laboratory methods in primatology. 2nd edition. Cambridge: Cambridge University Press.

Simmons AM, Popper AN, Fay RR, editors. 2003. Acoustic communication. New York: Springer.
